# Self-Assembled Lubricin (PRG-4)-Based Biomimetic Surface-Enhanced Raman Scattering Sensor for Direct Droplet Detection of Melamine in Undiluted Milk

**DOI:** 10.3390/bios14120591

**Published:** 2024-12-03

**Authors:** Mingyu Han, Mya Myintzu. Hlaing, Paul R. Stoddart, George W. Greene

**Affiliations:** 1 Commonwealth Scientific and Industrial Research Organization (CSIRO), Agriculture and Food, 671 Sneydes Road, Werribee, VIC 3030, Australia; myintzu.hlaing@csiro.au; 2 School of Science, Computing and Engineering Technologies, Swinburne University of Technology, Hawthorn, VIC 3122, Australia; pstoddart@swin.edu.au; 3 Department of Biochemistry and Chemistry, La Trobe University, Bundoora, VIC 3086, Australia; 4 La Trobe Institute for Molecular Science, La Trobe University, Bundoora, VIC 3086, Australia; 5 School of Agriculture, Biomedicine and Environment, La Trobe University, Bundoora, VIC 3086, Australia

**Keywords:** SERS, melamine, point-of-care, sensing, lubricin, AFM

## Abstract

Surface-enhanced Raman scattering (SERS) is a powerful optical sensing platform that amplifies the target signals by Raman scattering. Despite SERS enabling a meager detection limit, even at the single-molecule level, SERS also tends to equally enhance unwanted molecules due to the non-specific binding of noise molecules in clinical samples, which complicates its use in complex samples such as bodily fluids, environmental water, or food matrices. To address this, we developed a novel non-fouling biomimetic SERS sensor by self-assembling an anti-adhesive, anti-fouling, and size-selective Lubricin (LUB) coating on gold nanoparticle (AuNP) functionalized glass slide surfaces via a simple drop-casting method. Compared to a conventional AuNPs-SERS substrate, the biomimetic SERS meets the requirements of simple preparation and enables direct droplet detection without any sample pre-treatment. Atomic force microscopy was used to confirm the self-assembled Lubricin coating on the AuNP surface, acting as an anti-fouling and size-selective protection layer. A series of Raman spectra were collected using melamine as the target analyte, which was spiked into 150 mM NaCl solution or undiluted milk. It was demonstrated that the LUB coating effectively prevents the detrimental fouling generated by the proteins and fats in milk, ensuring the clear detection of melamine. Our sensor showed high selectivity and could detect melamine in milk at concentrations as low as 1 ppm. Given that the EU/US legal limit for melamine in food is 2.5 ppm, this sensor offers a promising, cost-effective solution for routine screening and has potential applications for detecting food adulteration in the food safety, environmental monitoring, aquaculture, and biomedical fields.

## 1. Introduction

Melamine, a nitrogen-rich organic compound, has been widely used in various industrial applications such as the manufacturing of dyes [1], inks [2], resins [3,4,5], coatings [5], and adhesives [6], after being polymerized with formaldehyde due to its stability and cost-effectiveness [7,8]. However, the improper use of melamine in the food industry, specifically in the milk and dairy sector, has garnered significant controversy because of its potential health risks and fraudulent use [9]. Melamine is illegally added to milk because it contains approximately 67% nitrogen by molecular weight. The practice of adding melamine to milk products was encouraged primarily by economic incentives. By artificially inflating the apparent protein content of milk, melamine allows unscrupulous producers to meet nutritional standards and pass quality tests, which typically use nitrogen content to estimate protein levels [7,10]. The primary drawback of using melamine in dairy products is its toxicological impact on human health [11,12,13,14,15,16]. When ingested, melamine reacts with cyanuric acid to form insoluble melamine–cyanurate crystals in the kidneys, triggering renal failure and other severe urinary complications [17,18,19,20,21]. The infamous 2008 Chinese milk scandal, which resulted in the hospitalization of tens of thousands of infants and several fatalities, starkly highlighted the dire consequences of melamine adulteration in food [22,23,24,25,26]. This incident underscored the health hazards associated with melamine and led to a global demand for stricter food safety regulations and more robust analytical methods to detect melamine levels in milk [27,28,29].

The detection and analysis of melamine contamination necessitate sophisticated analytical techniques, such as high-performance liquid chromatography [30,31,32,33], gas chromatography–mass spectrometry (GC-MS) [31,34], liquid chromatography–mass spectrometry (LC-MS) [31,35,36], and enzyme-linked immunosorbent assay (ELISA) [37,38,39,40,41], which are time-consuming, involve complex sample preparation and complicated operation procedures, and are relatively expensive. Non-destructive analytical methods such as electrochemical detection, infrared and Raman spectroscopy, and surface-enhanced Raman scattering (SERS) are considered better tools for providing rapid, accurate, and point-of-care (PoC) sensing results. Compared to other sensing techniques, SERS can provide fingerprint information of the target analyte, facilitating multiplex sensing results. SERS also yields high signal enhancement due to the localized surface plasmon resonance of metallic nanoparticles, e.g., Au nanoparticles [42,43,44,45]. Different SERS-based methods for melamine detection have been developed; most of them focus on improvements to SERS substrates such as fabricating new SERS substrates with high sensitivity and high performance [10,17,46,47,48,49], modifying the SERS substrate surface with SERS probes [50,51,52], and labeling metallic nanoparticles with SERS tags [53,54,55]. However, SERS analysis of target molecules in undiluted milk samples has not been addressed. Undiluted milk samples are complex mixtures of large-sized interference contaminants, including milk proteins like casein micelles (~0.3 µm), ions, and milk fat globules (ranging from 200 nm to 15 µm), with a range of absorptivity on the SERS active surface.

Lubricin (LUB) is a type of glycoprotein originally found in articular joints [56,57,58,59]. LUB can self-assemble on a variety of flat surfaces, including metallic, polymer, inorganic, 2D, and electrode surfaces [60,61,62,63,64,65,66,67,68]. The tri-block structure of the LUB molecule comprises two end-domains that adhere to the substrate surfaces and a 200 nm mucin domain carrying negative charges. When the LUB molecules adsorb on the substrate surface, the two highly adhesive end-domains bind to the substrate surface, while the heavily glycosylated central portion, named the mucin domain, forms a polymer-like brush due to the repulsive forces generated by negatively charged mucin domains. According to previous atomic force microscopy and neutron reflectometry studies [62,69,70], a fully extended LUB mucin domain forms a roughly 100 nm high, ‘telechelic’ structure. LUB coatings have been found to exhibit remarkable anti-adhesive, anti-fouling, and size-selective transport properties [60,63,70,71,72], performing even better than polyethylene glycol under certain conditions [63]. Compared to other polymeric anti-fouling layers, LUB coatings require no complex surface chemistry or engineering, thus preserving the surface properties of the sensor substrate. As a protective layer in electrochemical and optical (SERS) sensors, LUB coatings have been used to separate the target analyte from complex clinical samples such as saliva, protein solutions, undiluted milk, and unprocessed whole blood [71,72,73,74].

In this work, we investigate the use of LUB as a protective and separation layer in the SERS sensing platform for detecting melamine in undiluted milk samples. The self-assembled LUB-coated SERS sensor (biomimetic SERS) was fabricated for the rapid and sensitive detection of melamine in undiluted milk samples, and its sensing performance was compared with a bare SERS sensor (without the LUB protection layer). To confirm that Lubricin had self-assembled on the bottom of the AuNP surface, the AFM technique was used to characterize the bare SERS and biomimetic SERS and compare the morphologies and adhesion behaviors of the bare SERS and biomimetic SERS via AFM imaging and normal-force distance measurements, respectively. Compared to the bare SERS, the biomimetic SERS successfully detected undiluted milk samples spiked with melamine, with a detection limit of 1 ppm. This biomimetic SERS sensor demonstrates great potential for extension to various future applications, such as environmental contaminant analysis, food safety, and biofluid analysis.

## 2. Materials and Methods

### 2.1. Materials and Reagents

Melamine, H_2_O_2_, aminopropyltriethoxysilane (APTES, ≥98%), sodium tetrachloroaurate (III) dehydrate (AuCl_4_Na_2_·H_2_O), and sodium citrate tribasic dihydrate (Na_3_C_6_H_5_O_7_·2H_2_O) were purchased from Sigma-Aldrich, and H_2_SO_4_ was purchased from ASIS Scientific. Undiluted, ‘full cream’ milk was purchased from a local supermarket (Woolworths, Australia). The Lubricin (LUB), also known as PRG-4, utilized in this study was a recombinant human full-sequence variant with >99.5% purity, as verified and supplied by Lubris BioPharma (Framingham, MA, USA). The initial LUB concentration was 2.3 mg/mL in 1× PBS, with 0.1% polysorbate 20 added as a stabilizing agent, acting as both a surfactant and a potential adhesion enhancer for substrate surfaces. A total of 2.5 mL of LUB solution underwent dialysis against 500 mL of PBS without polysorbate using a Slide-A-Lyzer cassette with a 20,000 molecular weight cutoff. This process was conducted over 24 h, with the dialysate replaced after approximately 4 and 8 h. Once dialyzed, the LUB solution was divided into aliquots, flash-frozen in liquid nitrogen, and stored until needed. All chemicals were analytical-grade and used as received unless noted otherwise. Deionized water (DI water, 18.2 MΩ/cm) was provided by a Thermo Scientific Nanopure system (Waltham, MA, USA).

### 2.2. Sensor Fabrication

**Scheme 1:**
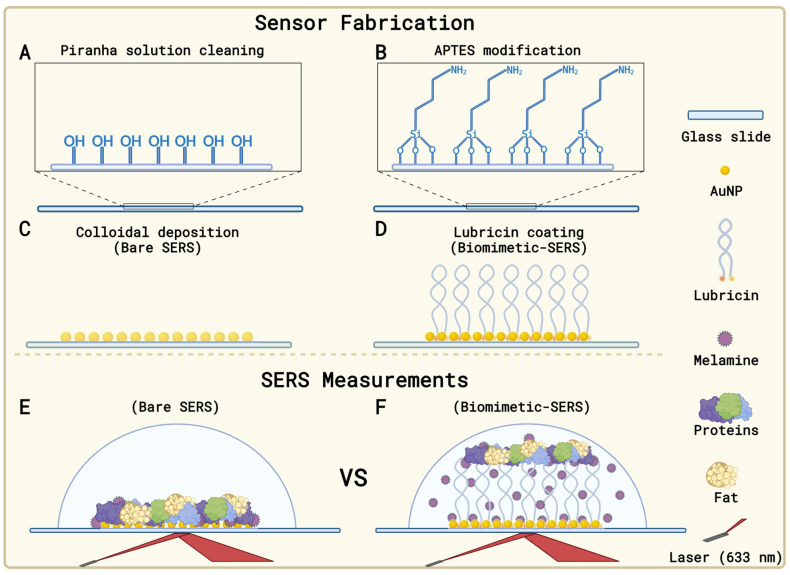
(**A**–**D**) Schematic illustration of the fabrication of the biomimetic SERS sensor. (**E**,**F**) SERS measurements of melamine-spiked undiluted milk with the bare SERS sensor and the biomimetic sensor, respectively.

The same Au nanoparticles (AuNPs) as reported in previous works were used to fabricate the easiest SERS active substrates [72,75]. Briefly, 40 mL of 1 mM stocked AuCl_4_Na_2_·H_2_O solution was heated to the boiling point, and then 4 mL of 1% sodium citrate solution was rapidly added to the AuCl_4_Na_2_·H_2_O solution while stirring. A colloidal Au solution gradually formed as the citrate reduced the gold (III), and the solution was kept heated until it turned deep red. To prepare the colloidal Au-modified surface (bare SERS), the glass slide was first rinsed with DI to remove any dust from the surface and then cleaned using a freshly prepared piranha solution, which consists of 98% H_2_SO_4_ and 30% H_2_O_2_ with a volume ratio of 3:1, for 0.5 h at 80 °C to remove any organic contaminations on the surface and obtain a completely smooth and hydroxylation layer, as shown in Scheme 1A. The cleaned glass slide was then modified with APTES to obtain a homogeneous amine group surface by the vapor deposited method. Specifically, the piranha solution-cleaned glass slide was put into a beaker with 1 mL of pure APTES and left in the oven overnight at 70 °C, followed by DI water rinse and curing at 110 °C for 15 min (see Scheme 1B). To obtain the AuNP-coated glass slide (Bare SERS), the glass slide was immersed in the gold colloidal solution for 2 h at room temperature and then the glass slide was rinsed with DI water, as shown in Scheme 1C. Finally, the Lubricin was self-assembled on the AuNP-coated glass slide to fabricate the biomimetic SERS by directly dropping 100 µL of 130 µg/mL stocked Lubricin solution for 20 min to allow the Lubricin molecules to adhere onto the AuNP-modified surface, which was then rinsed with a large amount of DI water to remove any unbound Lubricin molecules from the surface of the biomimetic SERS (see Scheme 1D). The drop-casting method has been proven to be an effective and efficient method in previous works using different surfaces as substrates, including Au surfaces [62,63,69,70,72,76], mica [62,68,77,78], polymers [62,79,80], 2D nanomaterials [61,62], and electrodes [60,71,73].

### 2.3. AFM Characterization of the Biomimetic SERS

The bare SERS and biomimetic SERS sensors were fabricated as above. The sensors were stored in Petri dishes containing PBS buffer for subsequent atomic force microscopy (AFM) imaging and normal-force distance measurements. As detailed in prior research [60,61,63,70,81], AFM imaging and measurements were performed using a JPK Nanowizard Sense AFM equipped with triangular silicon nitride cantilevers (Bruker MSCT). The cantilever ‘D’ from the MSCT multi-cantilever set, with a nominal tip radius of 10 nm and spring constant of 0.04 N/m, served as the imaging and measurement tip in contact mode. For normal-force distance measurements, a previously established protocol was employed. The cantilevers were first cleaned for 15 min using a biological UV–ozone cleaner to eliminate organic residues from the tips. Subsequently, the normal spring constant in air and the inverse optical lever sensitivity (invOLS) in 150 mM NaCl were calibrated. Measurements were performed with a ramp length of 2 µm, an approach and retraction velocity of 1 µm/s, and no dwell time at peak force. Data analysis was carried out using version 6.1.175 of the JPK Data Processing software.

## 3. Results

### 3.1. AFM Imaging and Normal Force Distance Measurements

The key component of the biomimetic SERS sensor is the self-assembled Lubricin layer on the bare SERS surface, which is expected to show the anti-fouling and size-selective properties when detecting melamine molecules in undiluted milk. AFM imaging and normal-force distance measurements were employed to confirm the existence of the LUB layer on the bare SERS surface. AFM imaging and normal-force distance measurements were performed to access the surface morphologies and adhesion behaviors, respectively, of the bare SERS and biomimetic SERS. The adhesion results of the LUB layer on the DNA sensor, including adhesion force, energy, and adhesion force range, provided information about the organization and uniformity of the LUB coatings, which are directly related to the anti-fouling property and sensing performance of the biomimetic DNA sensor.

**Figure 1:**
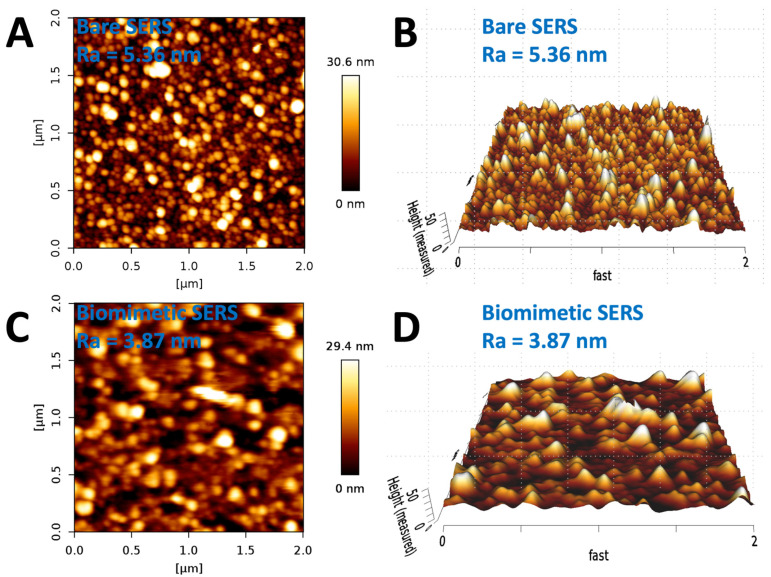
(**A**) Representative AFM images of bare SERS. (**B**) Corresponding 3D image of Figure 1A. (**C**) Representative AFM images of biomimetic SERS. (**D**) Corresponding 3D image of Figure 1C. The AFM images were collected using contact mode and the Ra values listed on the images are averages of the RMS data for each. The average roughness shown was calculated from ten total traces of an individual image.

The morphologies of the bare SERS and biomimetic SERS were measured via AFM imaging and are shown in Figure 1. Figure 1A shows the represented AFM images of bare SERS with an average roughness Ra value of 5.36 nm, and Figure 1B shows the 3D image of Figure 1A. Compared to the bare SERS, the biomimetic SERS (see Figure 1C,D) with an average roughness Ra value of 3.87 nm shows a noticeable decrease in surface roughness, indicating that LUB self-assembled on the SERS surface and formed a uniform layer, which is in agreement with our previously reported LUB-coated Au surface which was measured by the AFM imaging method [62,63,69,70,71,72].

**Figure 2:**
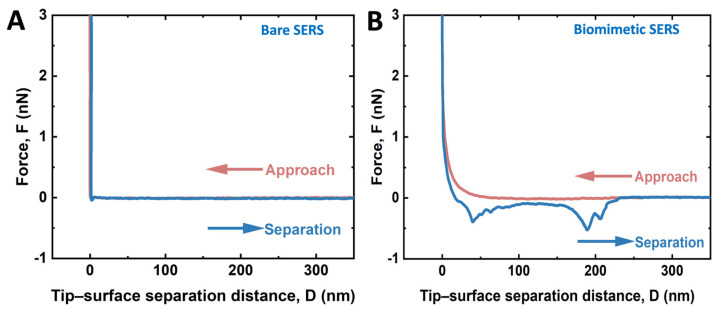
Representative AFM normal–force distance curves of (**A**) bare SERS and (**B**) biomimetic DNA sensor.

To further investigate the difference in the adhesion behaviors of bare SERS and biomimetic SERS, a series of AFM normal-force distance measurements were conducted on the bare SERS and biomimetic sensors and followed by plotting the normal-force distance curves of LUB on different surfaces, as shown in Figure 2. Figure 2 shows representative AFM normal-force distance measurements taken during the approach and separation of the AFM tip against the bare SERS and biomimetic sensor, respectively. For the normal-force distance curve of the bare SERS, as shown in Figure 2A and similar to what was previously reported [62,69,70], there was no adhesion measured on bare SERS since there was no LUB layer on the Au surface. In contrast, for the LUB-SERS plotted in Figure 2B, a typical normal-force distance force curve was obtained on the LUB-modified surface. Compared to the AFM normal-force distance curve of the bare SERS, a large increase in the range of the steric repulsive forces was measured during the approach stage due to the repulsive interactions between the AFM tip and the mucin domain of the LUB layer. During the separation process of Biomimetic SERS, there was an obvious adhesion behavior that occurred when the AFM tip was pulled up and away from the Biomimetic SERS surface. Briefly, the LUB molecule, bound to the AFM tip, was detached from the Biomimetic SERS in a fraction of the measurements, and the corresponding adhesion force and adhesion energy were recorded. The observed LUB adhesion force curve again indicates that the LUB molecules have self-assembled on the bare SERS surface and formed a ‘telechelic-like’ structure. 

### 3.2. Analysis of Melamine with Bare SERS

For the SERS measurements of buffer and undiluted milk media spiked with melamine, two types of sensors were used. The bare SERS sensor, fabricated by simply depositing colloidal Au nanoparticles on an APTES-modified glass microscope slide, is shown in Scheme 1C. The same bare SERS sensor showed good SERS activity for the detection of Rhodamine 6G- and L-cysteine-spiked buffer (150 mM NaCl) in a previous work [72]. Compared to the bare SERS sensor, the biomimetic SERS sensor was developed by coating a self-assembled Lubricin layer on the bare SERS sensor as a protection layer with anti-fouling and size-selective properties. The two end-domains of Lubricin physically adhere to the surface, while the 200 nm long, negatively charged mucin domain fully extends due to internal repulsive forces, forming a telechelic polymer-like loop structure on the substrate surface (see Scheme 1D) [61,62,68]. Previous works have shown that the self-assembled Lubricin layer covers only about 15% of the substrate surface area and consists of more than 95% water. The low coverage and highly diffuse nature of the Lubricin layer allow it to act as a molecular sieve, physically blocking large-sized fouling molecules such as proteins and cells, while small-sized target molecules, including ions, drugs, and other chemicals, can diffuse into the Lubricin layer and interact with the underlying substrate surface. The size-selective property of Lubricin has been demonstrated and utilized in electrochemical sensors [60,71,73] and optical (SERS) sensors [72].

**Figure 3:**
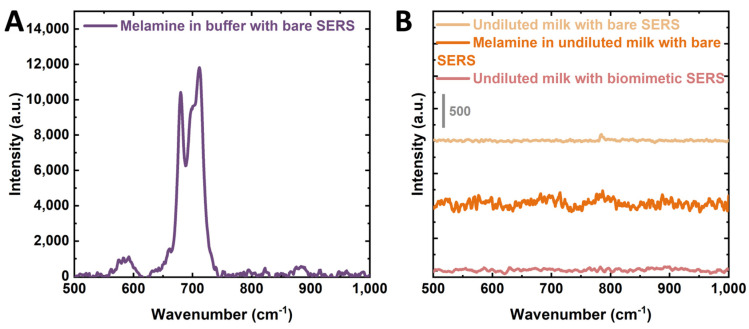
(**A**) SERS measurement of melamine in buffer made with bare SERS. (**B**) SERS measurements of pure milk and melamine–spiked undiluted milk made with the bare SERS sensor or biomimetic SERS, respectively.

Firstly, to obtain the SERS spectra of melamine in pure buffer as a reference, SERS measurements were conducted to analyze clean buffer (150 mM NaCl) spiked with melamine using the bare SERS (without the self-assembled Lubricin layer) as the sensor. Figure 3A displays the SERS spectra of the melamine-spiked buffer collected with bare SERS, in which the predominant peaks of the SERS spectra of melamine at 680 cm^−1^ and 700 cm^−1^, assigned to the triazine ring with a protonated form and a neutral molecular form, can be observed [46,82].

To understand the impact of the non-specific binding of fouling molecules such as fats and proteins in undiluted milk on the performance of bare SERS when used to detect melamine, the bare SERS sensor was next challenged with undiluted milk without the melamine target and with 100 ppm of the melamine target, respectively. Figure 3B shows the SERS spectra of undiluted milk only and melamine–spiked undiluted milk with the bare SERS sensor, respectively. There were no identical peaks observed for the undiluted milk. Similarly, as demonstrated in Scheme 1E, due to the uncontrolled non-specific bindings of unwanted fats and proteins in the undiluted milk, the target melamine molecules cannot access or bind on the bottom AuNPs, as a result of which there are no enhanced Raman signals from melamine generated at any position with the bare SERS sensor.

### 3.3. Analysis of Undiluted Milk Spiked with Melamine with Biomimetic SERS

**Figure 4:**
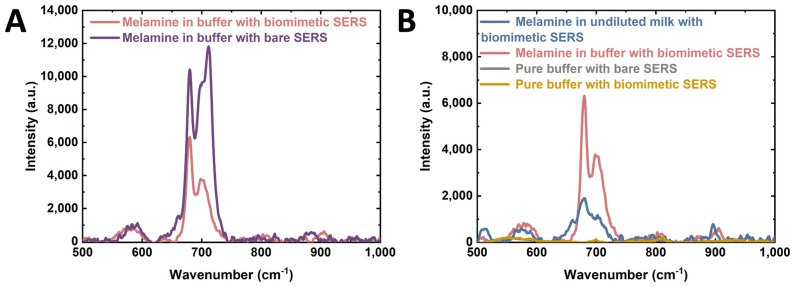
(**A**) SERS spectra of melamine–spiked buffer collected with biomimetic SERS and bare SERS. (**B**) SERS spectra of melamine–spiked undiluted milk or buffer with the same biomimetic SERS sensors and background spectra of bare SERS and biomimetic SERS without melamine.

Before evaluating the anti-fouling and size-selective transport properties of the self-assembled Lubricin layer, we studied the ability of target melamine molecules to diffuse into the Lubricin layer and adsorb onto the underlying AuNPs. SERS measurements were conducted using buffer spiked with 100 ppm melamine as the analyte, with bare SERS and biomimetic SERS used as the Raman sensors. Figure 4A shows the SERS spectra of melamine in buffer, obtained under identical measurement parameters using biomimetic SERS and bare SERS. Compared to the SERS spectra of melamine in buffer collected with bare SERS, the spectra collected using biomimetic SERS still show Raman scattering at the same peak positions. However, a noticeable decrease in peak intensities at both 680 cm^−1^ and 700 cm^−1^ is observed when the biomimetic SERS is used. Interestingly, the peak at 700 cm^−1^, attributed to the triazine ring with a neutral molecular form, was significantly affected by the self-assembled Lubricin layer. The decrease in peak intensity at 700 cm^−1^ may be due to the impact of the self-assembled Lubricin brush altering the orientation and form of the melamine molecules as the molecules diffused through the Lubricin brush layer to adsorb on the bottom of the AuNP surface. Despite the obvious effect of the self-assembled Lubricin layer on melamine as it travels through the layer, it can be concluded that the Lubricin layer does not significantly inhibit the ability of melamine molecules to access the AuNPs and generate SERS signals. 

To assess the anti-fouling and size-selective properties of the Lubricin layer in preventing the surface fouling of the sensor by the fats and proteins in the undiluted milk, the biomimetic SERS sensor was finally challenged against undiluted milk by directly pipetting the melamine-spiked undiluted milk sample onto the biomimetic SERS sensor’s surface. Figure 4B shows the SERS spectra of melamine collected from undiluted milk using the biomimetic SERS sensor and bare SERS, as well as the SERS spectra of melamine in a clean buffer collected with biomimetic SERS. Compared to the SERS spectra of melamine in buffer previously collected with biomimetic SERS, the overall intensity of the SERS spectra at 680 cm^−1^ is significantly reduced, a consequence of the reduction in ‘free’ melamine concentration due to the blocking of melamine molecules by milk proteins and fats. However, the SERS spectra of melamine are observed and identified, importantly maintaining the same peak position at 680 cm^−1^ with the biomimetic SERS sensor surface. There is a strong correlation between the melamine spectra collected in undiluted milk and clean buffer, both with a melamine concentration of 100 ppm. Again, comparing the melamine spectra of clean buffer and undiluted milk collected with biomimetic SERS and the spectra of undiluted milk collected with bare SERS confirms the Lubricin anti-fouling layer’s capability to identify melamine molecules from the complex food matrix, enabling its SERS detection.

### 3.4. Calibration of Undiluted Milk Spiked with Melamine with Biomimetic SERS

**Figure 5:**
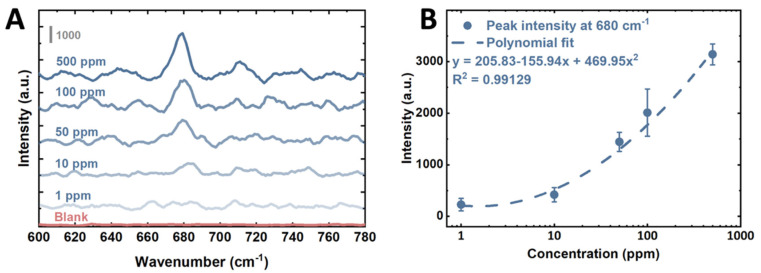
(**A**) SERS spectra under different concentrations of melamine in undiluted milk, ranging from 1 ppm to 500 ppm, were collected with biomimetic SERS. (**B**) Calibration curve of melamine peak intensity at 680 cm^–1^ as a function of melamine concentration in undiluted milk.

To evaluate the proportional relationship between melamine peak intensity and the concentration of melamine in undiluted milk, and to determine the limit of detection of the biomimetic SERS sensor for melamine in diluted milk, a calibration curve was generated using serial dilutions of melamine in milk until no further melamine peaks could be detected. Figure 5A shows representative melamine spectra collected using the biomimetic SERS sensor from serial dilutions of melamine-spiked undiluted milk, ranging from an initial concentration of 500 ppm to 1 ppm. The 680 cm^−1^ peak was chosen as the characteristic peak corresponding only to the melamine scattering and used to quantify the melamine concentration in milk. The relationship between the 680 cm^−1^ peak intensity and melamine concentration is shown in B. Based on the calibration curve in Figure 5B, the lowest detectable concentration of melamine in undiluted milk is 1 ppm.

## 4. Conclusions

In conclusion, the detection and analysis of melamine in undiluted milk were achieved by using the anti-fouling and size-selective transport properties of a self-assembled Lubricin layer, which was fabricated using a rapid and chemical reaction-free method. The Lubricin brush layer functions both as a protective layer, preventing fouling molecules like fats and milk proteins from contaminating the SERS surface, and as a size-selective membrane, allowing target molecules, such as melamine, to diffuse into the Lubricin layer and adsorb onto the AuNPs to generate SERS signals. The successful detection of melamine in undiluted milk strongly supports the application of Lubricin-covered sensing platforms for highly fouling food matrices like coffee and juice, aquaculture and environmental samples, and bodily fluids such as unprocessed whole blood, urine, and saliva for detecting and analyzing mycotoxins, antibiotics, DNA, drugs, and other clinically relevant analytes. Future work will focus on enhancing the selectivity of the sensor surface by employing target recognition techniques such as molecularly imprinted polymers. Besides developing analyte affinity, future research will investigate better SERS substrate surfaces to enhance the detection limit of the Lubricin-enabled biomimetic SERS sensor.

## Data Availability

All data in this research have been included in the main article.

## References

[1] Garg A.K., Singh B., Naskar S., Prajapati R.K., Dalal C., Sonkar S.K. (2023). Melamine–Formaldehyde Polymer-Based Nanocomposite for Sunlight-Driven Photodegradation of Multiple Dyes and Their Mixture. Langmuir.

[2] Kralj M., Krivačić S., Ivanišević I., Zubak M., Supina A., Marciuš M., Halasz I., Kassal P. (2022). Conductive Inks Based on Melamine Intercalated Graphene Nanosheets for Inkjet Printed Flexible Electronics. Nanomaterials.

[3] Yu Q., Bai J., Huang J., Demir M., Farghaly A.A., Aghamohammadi P., Hu X., Wang L. (2023). One-Pot Synthesis of Melamine Formaldehyde Resin-Derived N-Doped Porous Carbon for CO_2_ Capture Application. Molecules.

[4] Tian L., Zhi Y., Yu Q., Xu Q., Demir M., Colak S.G., Farghaly A.A., Wang L., Hu X. (2024). Enhanced CO_2_ Adsorption Capacity in Highly Porous Carbon Materials Derived from Melamine-Formaldehyde Resin. Energy Fuels.

[5] Dorieh A., Farajollah Pour M., Ghafari Movahed S., Pizzi A., Pouresmaeel Selakjani P., Valizadeh Kiamahalleh M., Hatefnia H., Shahavi M.H., Aghaei R. (2022). A review of recent progress in melamine-formaldehyde resin based nanocomposites as coating materials. Prog. Org. Coat..

[6] Jiang K., Lei Z., Yi M., Lv W., Jing M., Feng Q., Tan H., Chen Y., Xiao H. (2021). Improved performance of soy protein adhesive with melamine–urea–formaldehyde prepolymer. RSC Adv..

[7] Bischoff K., Gupta R.C. (2019). Chapter 31-Melamine. Biomarkers in Toxicology.

[8] Bann B., Miller S.A. (1958). Melamine And Derivatives Of Melamine. Chem. Rev..

[9] Tyan Y.-C., Yang M.-H., Jong S.-B., Wang C.-K., Shiea J. (2009). Melamine contamination. Anal. Bioanal. Chem..

[10] Li L., Chin W.S. (2021). Rapid and sensitive SERS detection of melamine in milk using Ag nanocube array substrate coupled with multivariate analysis. Food Chem..

[11] Skinner C.G., Thomas J.D., Osterloh J.D. (2010). Melamine Toxicity. J. Med. Toxicol..

[12] Rajpoot M., Bhattacharya R., Sharma S., Gupta S., Sharma V., Sharma A.K. (2021). Melamine contamination and associated health risks: Gut microbiota does make a difference. Biotechnol. Appl. Biochem..

[13] Li Q., Song P., Wen J. (2019). Melamine and food safety: A 10-year review. Curr. Opin. Food Sci..

[14] Liu S., Zhao Q., Huang F., Yang Q., Wang Y., Wang H., Sun Y., Yan Y., He G., Zhao G. (2022). Exposure to melamine and its derivatives in Chinese adults: The cumulative risk assessment and the effect on routine blood parameters. Ecotoxicol. Environ. Saf..

[15] Shi X., Dong R., Chen J., Yuan Y., Long Q., Guo J., Li S., Chen B. (2020). An assessment of melamine exposure in Shanghai adults and its association with food consumption. Environ. Int..

[16] Melough M.M., Foster D., Fretts A.M., Sathyanarayana S. (2020). Dietary Sources of Melamine Exposure among US Children and Adults in the National Health and Nutrition Examination Survey 2003–2004. Nutrients.

[17] Xiao G., Li L., Yan A., He X. (2019). Direct detection of melamine in infant formula milk powder solution based on SERS effect of silver film over nanospheres. Spectrochim. Acta Part A Mol. Biomol. Spectrosc..

[18] Hau A.K.-C., Kwan T.H., Li P.K.-T. (2009). Melamine Toxicity and the Kidney. J. Am. Soc. Nephrol..

[19] Zhu H., Kannan K. (2019). Melamine and cyanuric acid in foodstuffs from the United States and their implications for human exposure. Environ. Int..

[20] Abedini R., Khaniki G.J., Naderi M., Aghaee E.M., Sadighara P. (2023). Investigation of melamine and cyanuric acid concentration in several brands of liquid milk and its non-carcinogenic risk assessment in adults and infants. J. Food Sci. Technol..

[21] Liu C.-C., Hsieh T.-J., Wu C.-F., Lee C.-H., Tsai Y.-C., Huang T.-Y., Wen S.-C., Lee C.-H., Chien T.-M., Lee Y.-C. (2020). Interrelationship of environmental melamine exposure, biomarkers of oxidative stress and early kidney injury. J. Hazard. Mater..

[22] Gossner Céline M.-E., Schlundt J., Ben Embarek P., Hird S., Lo-Fo-Wong D., Beltran Jose Javier O., Teoh Keng N., Tritscher A. (2009). The Melamine Incident: Implications for International Food and Feed Safety. Environ. Health Perspect..

[23] Li S., Wang Y., Tacken G.M.L., Liu Y., Sijtsema S.J. (2021). Consumer trust in the dairy value chain in China: The role of trustworthiness, the melamine scandal, and the media. J. Dairy Sci..

[24] Yang R., Huang W., Zhang L., Thomas M., Pei X. (2009). Milk adulteration with melamine in China: Crisis and response. Qual. Assur. Saf. Crops Foods.

[25] Qiao G., Guo T., Klein K.K. (2010). Melamine in Chinese milk products and consumer confidence. Appetite.

[26] Qiao G., Guo T., Klein K.K. (2012). Melamine and other food safety and health scares in China: Comparing households with and without young children. Food Control.

[27] Smulders F.J.M., Rietjens I.M.C.M., Rose M. (2023). Chemical Hazards in Foods of Animal Origin.

[28] Pei X., Tandon A., Alldrick A., Giorgi L., Huang W., Yang R. (2011). The China melamine milk scandal and its implications for food safety regulation. Food Policy.

[29] Chan E.Y.Y., Griffiths S.M., Chan C.W. (2008). Public-health risks of melamine in milk products. Lancet.

[30] Venkatasami G., Sowa J.R. (2010). A rapid, acetonitrile-free, HPLC method for determination of melamine in infant formula. Anal. Chim. Acta.

[31] Lutter P., Savoy-Perroud M.-C., Campos-Gimenez E., Meyer L., Goldmann T., Bertholet M.-C., Mottier P., Desmarchelier A., Monard F., Perrin C. (2011). Screening and confirmatory methods for the determination of melamine in cow’s milk and milk-based powdered infant formula: Validation and proficiency-tests of ELISA, HPLC-UV, GC-MS and LC-MS/MS. Food Control.

[32] Qin Z., Jiang Y., Piao H., Li J., Tao S., Ma P., Wang X., Song D., Sun Y. (2020). MIL-101(Cr)/MWCNTs-functionalized melamine sponges for solid-phase extraction of triazines from corn samples, and their subsequent determination by HPLC-MS/MS. Talanta.

[33] Öztürk S., Demir N. (2021). Development of a novel IMAC sorbent for the identification of melamine in dairy products by HPLC. J. Food Compos. Anal..

[34] Pan X.-D., Wu P.-G., Yang D.-J., Wang L.-Y., Shen X.-H., Zhu C.-Y. (2013). Simultaneous determination of melamine and cyanuric acid in dairy products by mixed-mode solid phase extraction and GC–MS. Food Control.

[35] Wu C.-F., Cheng C.-M., Hsu Y.-M., Li S.-S., Huang C.-Y., Chen Y.-H., Kuo F.-C., Wu M.-T. (2020). Development of analytical method of melamine in placenta from pregnant women by isotope-dilution liquid chromatography/tandem mass spectrometry. Rapid Commun. Mass Spectrom..

[36] Zhu H., Kannan K. (2020). Determination of melamine and its derivatives in textiles and infant clothing purchased in the United States. Sci. Total Environ..

[37] Yin W., Liu J., Zhang T., Li W., Liu W., Meng M., He F., Wan Y., Feng C., Wang S. (2010). Preparation of Monoclonal Antibody for Melamine and Development of an Indirect Competitive ELISA for Melamine Detection in Raw Milk, Milk Powder, and Animal Feeds. J. Agric. Food Chem..

[38] Garber E.A.E. (2008). Detection of Melamine Using Commercial Enzyme-Linked Immunosorbent Assay Technology. J. Food Prot..

[39] Pan B., He Q., Yu X., De Choch D., Lam K.S., Hammock B.D., Sun G. (2024). Versatility and stability of melamine foam-based biosensors (f-ELISA) using antibodies, nanobodies, and peptides as sensing probes. Talanta.

[40] Han M., Gong L., Wang J., Zhang X., Jin Y., Zhao R., Yang C., He L., Feng X., Chen Y. (2019). An octuplex lateral flow immunoassay for rapid detection of antibiotic residues, aflatoxin M1 and melamine in milk. Sens. Actuators B Chem..

[41] Chen Q., Qie M., Peng X., Chen Y., Wang Y. (2020). Immunochromatographic assay for melamine based on luminescent quantum dot beads as signaling probes. RSC Adv..

[42] Han X.X., Rodriguez R.S., Haynes C.L., Ozaki Y., Zhao B. (2022). Surface-enhanced Raman spectroscopy. Nat. Rev. Methods Primers.

[43] Garrell R.L. (1989). Surface-enhanced Raman spectroscopy. Anal. Chem..

[44] Kneipp K., Kneipp H., Itzkan I., Dasari R.R., Feld M.S. (1999). Ultrasensitive chemical analysis by Raman spectroscopy. Chem. Rev..

[45] Silly F., Shaw A.Q., Castell M.R., Briggs G.A.D., Mura M., Martsinovich N., Kantorovich L. (2008). Melamine Structures on the Au(111) Surface. J. Phys. Chem. C.

[46] Zhang C., You T., Yang N., Gao Y., Jiang L., Yin P. (2019). Hydrophobic paper-based SERS platform for direct-droplet quantitative determination of melamine. Food Chem..

[47] Yang Z., Ma C., Gu J., Wu Y., Zhu C., Li L., Gao H., Yin W., Wang Z., Chen G. (2023). Detection of melamine by using carboxyl-functionalized Ag-COF as a novel SERS substrate. Food Chem..

[48] Huang C., Jiang S., Kou F., Guo M., Li S., Yu G., Zheng B., Xie F., Zhang C., Yu H. (2022). Development of jellyfish-like ZnO@Ag substrate for sensitive SERS detection of melamine in milk. Appl. Surf. Sci..

[49] Liu S., Kannegulla A., Kong X., Sun R., Liu Y., Wang R., Yu Q., Wang A.X. (2020). Simultaneous colorimetric and surface-enhanced Raman scattering detection of melamine from milk. Spectrochim. Acta Part A Mol. Biomol. Spectrosc..

[50] Lou T., Wang Y., Li J., Peng H., Xiong H., Chen L. (2011). Rapid detection of melamine with 4-mercaptopyridine-modified gold nanoparticles by surface-enhanced Raman scattering. Anal. Bioanal. Chem..

[51] Zhuang H., Zhu W., Yao Z., Li M., Zhao Y. (2016). SERS-based sensing technique for trace melamine detection–A new method exploring. Talanta.

[52] Sharma V., Som N.N., Pillai S.B., Jha P.K. (2020). Utilization of doped GQDs for ultrasensitive detection of catastrophic melamine: A new SERS platform. Spectrochim. Acta Part A Mol. Biomol. Spectrosc..

[53] Yazgan N.N., Boyacı İ.H., Topcu A., Tamer U. (2012). Detection of melamine in milk by surface-enhanced Raman spectroscopy coupled with magnetic and Raman-labeled nanoparticles. Anal. Bioanal. Chem..

[54] Giovannozzi A.M., Rolle F., Sega M., Abete M.C., Marchis D., Rossi A.M. (2014). Rapid and sensitive detection of melamine in milk with gold nanoparticles by Surface Enhanced Raman Scattering. Food Chem..

[55] Nie B., Luo Y., Shi J., Gao L., Duan G. (2019). Bowl-like Pore array made of hollow Au/Ag alloy nanoparticles for SERS detection of melamine in solid milk powder. Sens. Actuators B Chem..

[56] Jay G.D. (2004). Lubricin and surfacing of articular joints. Curr. Opin. Orthop..

[57] Jay G.D., Harris D.A., Cha C.-J. (2001). Boundary lubrication by lubricin is mediated by O-linked β(1-3)Gal-GalNAc oligosaccharides. Glycoconj. J..

[58] Ikegawa S., Sano M., Koshizuka Y., Nakamura Y. (2000). Isolation, characterization and mapping of the mouse and human PRG4 (proteoglycan 4) genes. Cytogenet. Genome Res..

[59] Swann D.A., Silver F.H., Slayter H.S., Stafford W., Shore E. (1985). The molecular structure and lubricating activity of lubricin isolated from bovine and human synovial fluids. Biochem. J..

[60] Russo M.J., Han M.Y., Quigley A.F., Kapsa R.M.I., Moulton S.E., Doeven E., Guijt R., Silva S.M., Greene G.W. (2020). Lubricin (PRG4) reduces fouling susceptibility and improves sensitivity of carbon-based electrodes. Electrochim. Acta.

[61] Han M., Berry J.D., Silva S.M., Vidallon M.L.P., Lei W., Quigley A.F., Kapsa R.M.I., Moulton S.E., Tabor R., Greene G.W. (2020). Self-Assembly of Lubricin (PRG-4) Brushes on Graphene Oxide Affords Stable 2D-Nanosheets in Concentrated Electrolytes and Complex Fluids. ACS Appl. Nano Mater..

[62] Han M., Silva S.M., Lei W., Quigley A., Kapsa R.M.I., Moulton S.E., Greene G.W. (2019). Adhesion and Self-Assembly of Lubricin (PRG4) Brush Layers on Different Substrate Surfaces. Langmuir.

[63] Greene G.W., Martin L.L., Tabor R.F., Michalczyk A., Ackland L.M., Horn R. (2015). Lubricin: A versatile, biological anti-adhesive with properties comparable to polyethylene glycol. Biomaterials.

[64] Aninwene G.E., Abadian P.N., Ravi V., Taylor E.N., Hall D.M., Mei A., Jay G.D., Goluch E.D., Webster T.J. (2015). Lubricin: A novel means to decrease bacterial adhesion and proliferation. J. Biomed. Mater. Res. Part A.

[65] Chang D.P., Guilak F., Jay G.D., Zauscher S. (2014). Interaction of lubricin with type II collagen surfaces: Adsorption, friction, and normal forces. J. Biomech..

[66] Schmidt T.A., Sullivan D.A., Knop E., Richards S.M., Knop N., Liu S., Sahin A., Darabad R.R., Morrison S., Kam W.R. (2013). Transcription, translation, and function of lubricin, a boundary lubricant, at the ocular surface. JAMA Ophthalmol..

[67] Subbaraman L.N., Schmidt T.A., Sheardown H. (2012). Proteoglycan 4 (lubricin) Enhances the Wettability of Model Conventional And Silicone Hydrogel Contact Lenses. Investig. Ophthalmol. Vis. Sci..

[68] Zappone B., Ruths M., Greene G.W., Jay G.D., Israelachvili J.N. (2007). Adsorption, Lubrication, and Wear of Lubricin on Model Surfaces: Polymer Brush-Like Behavior of a Glycoprotein. Biophys. J..

[69] Han M., Russo M.J., Desroches P.E., Silva S.M., Quigley A.F., Kapsa R.M.I., Moulton S.E., Greene G.W. (2024). Calcium ions have a detrimental impact on the boundary lubrication property of hyaluronic acid and lubricin (PRG-4) both alone and in combination. Colloids Surf. B Biointerfaces.

[70] Ye H., Han M., Huang R., Schmidt T.A., Qi W., He Z., Martin L.L., Jay G.D., Su R., Greene G.W. (2019). Interactions between Lubricin and Hyaluronic Acid Synergistically Enhance Antiadhesive Properties. ACS Appl. Mater. Interfaces.

[71] Greene G.W., Ortiz V., Pozo-Gonzalo C., Moulton S.E., Wang X., Martin L.L., Michalczky A., Howlett P.C. (2018). Lubricin Antiadhesive Coatings Exhibit Size-Selective Transport Properties that Inhibit Biofouling of Electrode Surfaces with Minimal Loss in Electrochemical Activity. Adv. Mater. Interfaces.

[72] Han M., Silva S.M., Russo M.J., Desroches P.E., Lei W., Quigley A.F., Kapsa R.M.I., Moulton S.E., Stoddart P.R., Greene G.W. (2024). Lubricin (PRG-4) anti-fouling coating for surface-enhanced Raman spectroscopy biosensing: Towards a hierarchical separation system for analysis of biofluids. Analyst.

[73] Li M., Abeyrathne C., Langley D.P., Cossins L.R., Samudra A.N., Green G.W., Moulton S.E., Silva S.M. (2023). Highly specific lubricin-lectin electrochemical sensor for glycoprotein cancer biomarker detection. Electrochim. Acta.

[74] Silva S.M., Langley D.P., Cossins L.R., Samudra A.N., Quigley A.F., Kapsa R.M.I., Tothill R.W., Greene G.W., Moulton S.E. (2022). Rapid Point-of-Care Electrochemical Sensor for the Detection of Cancer Tn Antigen Carbohydrate in Whole Unprocessed Blood. ACS Sens..

[75] Hou T., Martin L.L., Horn R.G., Greene G.W. (2016). Use of optical interferometry to measure gold nanoparticle adsorption on silica. Colloids Surf. A Physicochem. Eng. Asp..

[76] Russo M.J., Han M., Desroches P.E., Manasa C.S., Dennaoui J., Quigley A.F., Kapsa R.M.I., Moulton S.E., Guijt R.M., Greene G.W. (2021). Antifouling Strategies for Electrochemical Biosensing: Mechanisms and Performance toward Point of Care Based Diagnostic Applications. ACS Sens..

[77] Huang J., Qiu X., Xie L., Jay G.D., Schmidt T.A., Zeng H. (2019). Probing the Molecular Interactions and Lubrication Mechanisms of Purified Full-length Recombinant Human Proteoglycan 4 (rhPRG4) and Hyaluronic Acid (HA). Biomacromolecules.

[78] Chang D.P., Abu-Lail N.I., Coles J.M., Guilak F., Jay G.D., Zauscher S. (2009). Friction Force Microscopy of Lubricin and Hyaluronic Acid between Hydrophobic and Hydrophilic Surfaces. Soft Matter.

[79] Samsom M., Iwabuchi Y., Sheardown H., Schmidt T.A. (2018). Proteoglycan 4 and hyaluronan as boundary lubricants for model contact lens hydrogels. J. Biomed. Mater. Research. Part B Appl. Biomater..

[80] Ma Y., Cao X., Feng X., Ma Y., Zou H. (2007). Fabrication of super-hydrophobic film from PMMA with intrinsic water contact angle below 90°. Polymer.

[81] Russo M.J., Han M., Menon N.G., Quigley A.F., Kapsa R.M.I., Moulton S.E., Guijt R.M., Silva S.M., Schmidt T.A., Greene G.W. (2022). Novel Boundary Lubrication Mechanisms from Molecular Pillows of Lubricin Brush-Coated Graphene Oxide Nanosheets. Langmuir.

[82] Mircescu N.E., Oltean M., Chiş V., Leopold N. (2012). FTIR, FT-Raman, SERS and DFT study on melamine. Vib. Spectrosc..

